# Individual tree segmentation from UAV LiDAR data based on PointNet++ network

**DOI:** 10.3389/fpls.2026.1985967

**Published:** 2026-10-28

**Authors:** Haoran Liu, Zongling Jiang, Qiang Wang

**Affiliations:** College of Surveying and Mapping Engineering, Heilongjiang Institute of Technology, Harbin, China

**Keywords:** deep learning, point cloud, PointNet++, tree segmentation, UAV lidar

## Abstract

Accurately identifying and extracting individual trees is an important foundational task in conducting forest inventory using remote sensing techniques. In this study, the common mixed coniferous and broadleaf forest in the northeastern region of China was selected as the study area, and the point cloud data was obtained using an unmanned aerial vehicle (UAV)-based light detection and ranging (LiDAR) system. In order to improve the accuracy of individual tree segmentation in mixed forest, an individual tree segmentation method based on the PointNet++ deep learning network was proposed. Firstly, a training dataset that included multi-channel features such as coordinates, RGB, intensity, and echo count was constructed. The channel attention mechanism and Inverted Residual Multilayer Perceptron module were added to the original PointNet++ network model to enhance the performance of the network. Then, the network was used to perform semantic segmentation on the coniferous trees, broadleaf trees, and ground points in the mixed forest. Based on the results of semantic segmentation, a method that combined local highest points and density maximum points to locate the individual trees was proposed and individual tree segmentation was finally achieved through a layer-stacking segmentation algorithm. Comparative analysis with three other traditional segmentation algorithms (Marker-based watershed algorithm, distance-based segmentation algorithm, mean shift algorithm, and PointNet) showed that the improved PointNet++ achieved the highest accuracy among the compared methods in this study (*r* = 0.836, *p* = 0.849, *F*1 = 0.843). Under the experimental conditions of this study, the proposed method improved the segmentation accuracy of individual trees in the mixed coniferous and broadleaf forest and can provide basic data for subsequent studies on tree structure parameter extraction, species identification, and biomass estimation.

## 1 Introduction

Forests are the most widely distributed ecosystem on land, which are characterized by complex structural composition and rich biodiversity (Hu., 2021). Individual trees are the fundamental elements of forest ecosystems, whose spatial structure, growth status, and species distribution are key factors required for forest resource surveys and ecological environment modeling (Wolf et al., 2012; Fan et al., 2022). However, traditional tree measurements typically require fieldwork, which is time-consuming, inefficient, and costly (Zhang et al., 2015). As an active remote sensing method, Light Detection and Ranging (LiDAR) can simultaneously capture the horizontal and vertical structure characteristics of scanned objects, providing a new technological means for rapid and accurate tree information acquisition compared to imagery data (Jaakkola et al., 2010). LiDAR scanning systems are portable and can be mounted on various platforms such as cars, aircraft, satellites, and ground stations (Li et al., 2016). In addition, the rapid development of Unmanned Aerial Vehicle (UAV) technology has provided a novel platform for LiDAR data collection. UAV platforms have advantages such as high flexibility, strong operability, and high efficiency, while also offering denser three-dimensional point clouds (Tang et al., 2023; Chen et al., 2023).

Individual tree segmentation is a fundamental step in extracting tree structural parameters and classifying tree species, individual tree segmentation methods in forestry can generally be divided into two categories: (1) Based on the canopy height model (CHM). The CHM is a model that reflects the vertical distance between the canopy and the ground, usually obtained through the rasterization of point clouds. Common segmentation algorithms include local maximum methods (Duncanson et al., 2014), watershed algorithms (Jakubowski et al., 2013), and graph-based segmentation methods (Strîmbu and Strîmbu, 2015). These methods generally do not rely on prior knowledge, making them highly advantageous for tree segmentation in expansive forest areas. Koch et al. (2006) used a local maximum filter to detect tree crown apex positions in rasterized point cloud data and then applied watershed algorithms to achieve segmentation. Chen (2007) proposed to add the variable window in the canopy maxima model to search for local maxima and applied the marker-controlled watershed algorithm for tree segmentation. Liu et al. (2019) developed a multi-scale local maximum algorithm to identify tree crown apex positions (i.e., seed points) from CHMs at different resolutions. Then, trees were further separated by applying vertical profile analysis to the seed points. CHMs can simplify 3D data by reducing its dimensionality and complexity, enhancing algorithm efficiency when processing large-scale datasets. However, the interpolation and smoothing steps in CHM generation can introduce random errors or cause information loss, ultimately reducing the accuracy of individual tree segmentation. (2) Based on discrete point clouds. Direct processing of point cloud data helps minimize information loss when identifying individual trees. Common methods include the K-means clustering algorithm (Liu et al., 2022), the Mean shift algorithm (Duong et al., 2016), and the voxel spatial projection method (Lee et al., 2010). Ma et al. (2020) integrated spatial information such as tree region growth and canopy morphological structure to determine the number of trees using the correlation coefficients between canopy structures. Furthermore, the K-means algorithm was used to extract the individual tree point clouds. Yan et al. (2018) utilized Mean shift algorithm to perform coarse segmentation of tree point clouds obtained by UAV LiDAR. Then, the normalized segmentation algorithm was performed for secondary iterative segmentation in under-segmentation areas until individual trees were accurately identified. Ayrey et al. (2017) introduced a layer-stacking algorithm in the vertical direction of trees to slice the point cloud data into layers. The contours of trees were extracted and merged in each layer to ultimately obtain complete individual tree. These studies demonstrate that the direct segmentation of individual trees based on point cloud data can effectively utilize the vertical structural characteristics of trees. It has become the mainstream approach for tree segmentation. However, the large volume of point cloud data can significantly increase the time cost, resulting in a reduction of segmentation efficiency. Especially in mixed coniferous and broadleaf forest with complex and variable structures, the segmentation effectiveness of these methods for individual trees still needs to be improved.

With the development of artificial intelligence technology, deep learning has gradually become one of the most widely used methods for big data processing and analysis. Deep learning-based algorithms have shown strong generalization capabilities in handling 3D point cloud data (Liu et al., 2023). PointNet (Qi et al., 2017a) was the first deep network to directly process unordered point clouds using shared MLPs and max pooling, but it lacks local feature aggregation. PointNet++ (Qi et al., 2017b) addresses this limitation by hierarchical set abstraction (SA) layers that capture local structures at multiple scales. Although deep learning has made some progress in tree segmentation, most relevant studies focused on plantations, nurseries, and urban forest, with relatively simple structural composition (Li., 2023). Therefore, the practical application potential of deep learning in complex forest stands remains to be further explored.

In this study, a mixed coniferous and broadleaf forest located in Harbin Experimental Forest Farm, Shangzhi City, Heilongjiang Province, China was selected as the research object. Various segmentation algorithms were applied to perform individual tree segmentation, and the results were compared to identify suitable methods for complex forest environments. The objectives of this study are to: (1) Propose an individual tree segmentation algorithm for mixed coniferous and broadleaf forest based on PointNet++. (2) Compare the performance of the proposed algorithm with traditional segmentation algorithms. (3) Evaluate the effects of different influencing factors on individual tree segmentation.

## 2 Materials

The study area was located in Mao’er Mountain Experimental Forest Farm, Shangzhi City, Heilongjiang Province, China (127°18′0″〜127°41′6″E, 45°2′20″〜45°18′16″N). The topography of Mao’er Mountain Experimental Forest Farm is undulating, and the distribution of tree species is not uniform. The study plots were established in a gently sloping valley location which had rich and homogeneous tree species. About 7.5 hectares of plots were divided using the 50 m × 50 m grid. The vegetation in the plots was typical in the study area.

In this study, the point cloud data collection method was the same as that of the previous reference (Liu et al., 2024). The point cloud of the study area consisted of 114 million points with an average point density of 200 pts m^2^. Based on high-resolution aerial imagery, each point in the point cloud data was assigned RGB values in three channels. The extracted point cloud was stored in LAS 1.2 format. Field measurement data were collected on July 31, 2022. The random sampling method was used to obtain 15 plots with 50 m × 50 m from the grid of the study area. All trees within each plot were individually measured and recorded for their essential information, as shown in **Table 1**. The locations of the trees were marked using Real-time Kinematic (RTK, Model Qianxunxingyao X), which was manufactured by Qianxun Spatial Intelligence Inc. The location of the plots is shown in **Figure 1**.

**Table 1:** Field measurement statistics of six tree species across 15 sample plots in the Mao’er Mountain study area.

Tree species	Number	DBH	Height	Crown width
*Larix gmelinii*	275	17.2cm	17.6m	3.6m
*Pinus koraiensis*	312	17.7cm	14.2m	4.4m
*Ulmus pumila*	364	32.3cm	19.6m	13.2m
*Fraxinus mandshurica*	246	26.8cm	20.1m	9.4m
*Juglans mandshurica*	227	28.4cm	19.3m	9.1m
*Betula platyphylla*	282	18.9cm	18.8m	5.7m

**Figure 1:**
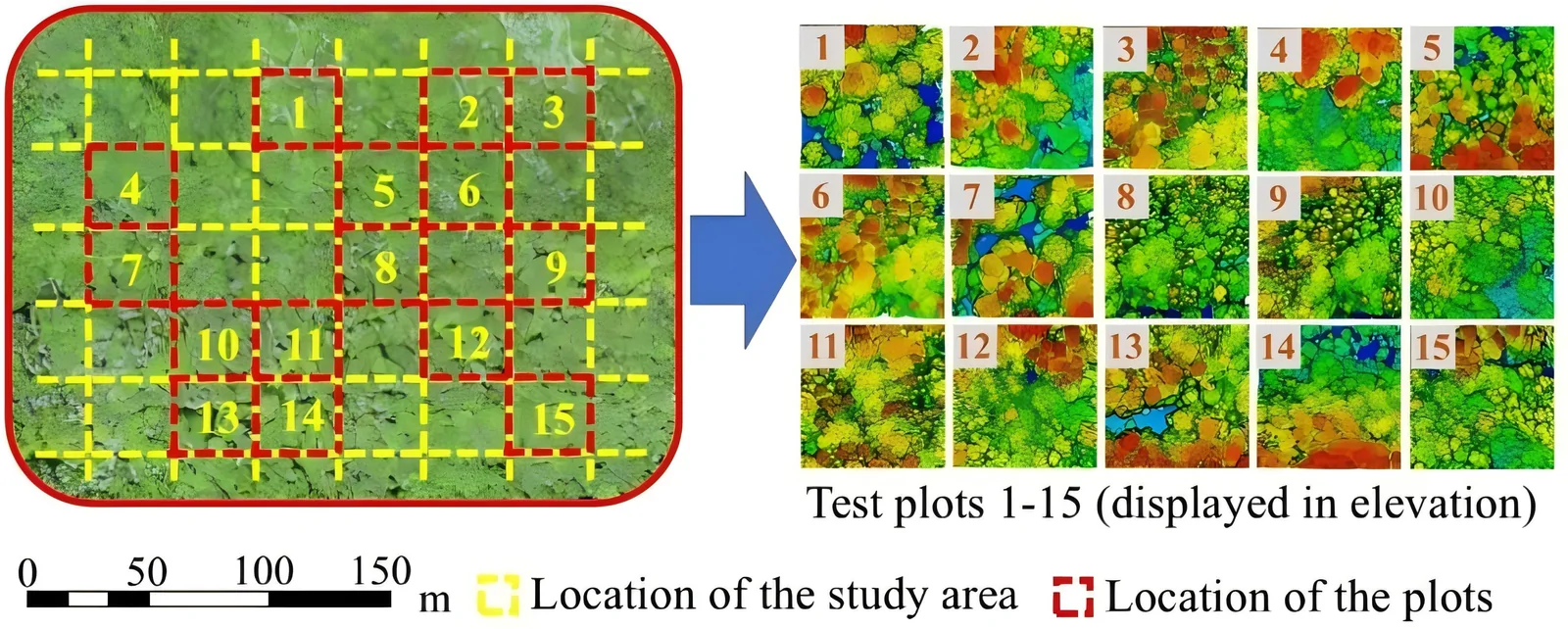
Location of the study area and plots.

## 3 Methods

### 3.1 Individual tree segmentation based on PointNet++

#### 3.1.1 Data preparation

The significant differences in crown shape and volume between coniferous and broadleaf trees often have negative impacts on parameter settings and the accuracy of individual tree segmentation algorithms. Therefore, the semantic segmentation of different tree types (coniferous and broadleaf) and ground points is helpful in simplifying the complex segmentation task, thereby enabling more accurate individual tree segmentation. The deep learning network PointNet++ was used for semantic segmentation of the experimental plots in mixed coniferous and broadleaf forest. The training and test sets were split at the plot level. Among the 15 field plots, 10 plots were used for training, and the remaining 5 plots were held out for testing. No points, trees, or patches from the test plots were used during training. Due to the limited number of plots, an exact 7:3 plot-level split was not feasible. The 10 training plots and 5 test plots yielded a training-to-test ratio of approximately 7:3 based on the number of individual trees. Different categories of point cloud data are shown in **Figure 2**. In addition, the basic input information of the PointNet++ network was the point cloud coordinates (X, Y, Z), which were difficult to effectively segment for complex and variable tree point clouds. Therefore, the segmentation accuracy by integrating other features, including RGB information (R, G, B), laser intensity (I), and echo counts (N) was improved. After obtaining the coordinates of individual tree points, multi-feature information was integrated to construct a multidimensional point set -- (X, Y, Z; R, G, B; I; N). The feature information was then normalized to the range of [-1, 1]. Subsequently, different class labels were assigned to each point in the training set and initialized.

**Figure 2:**
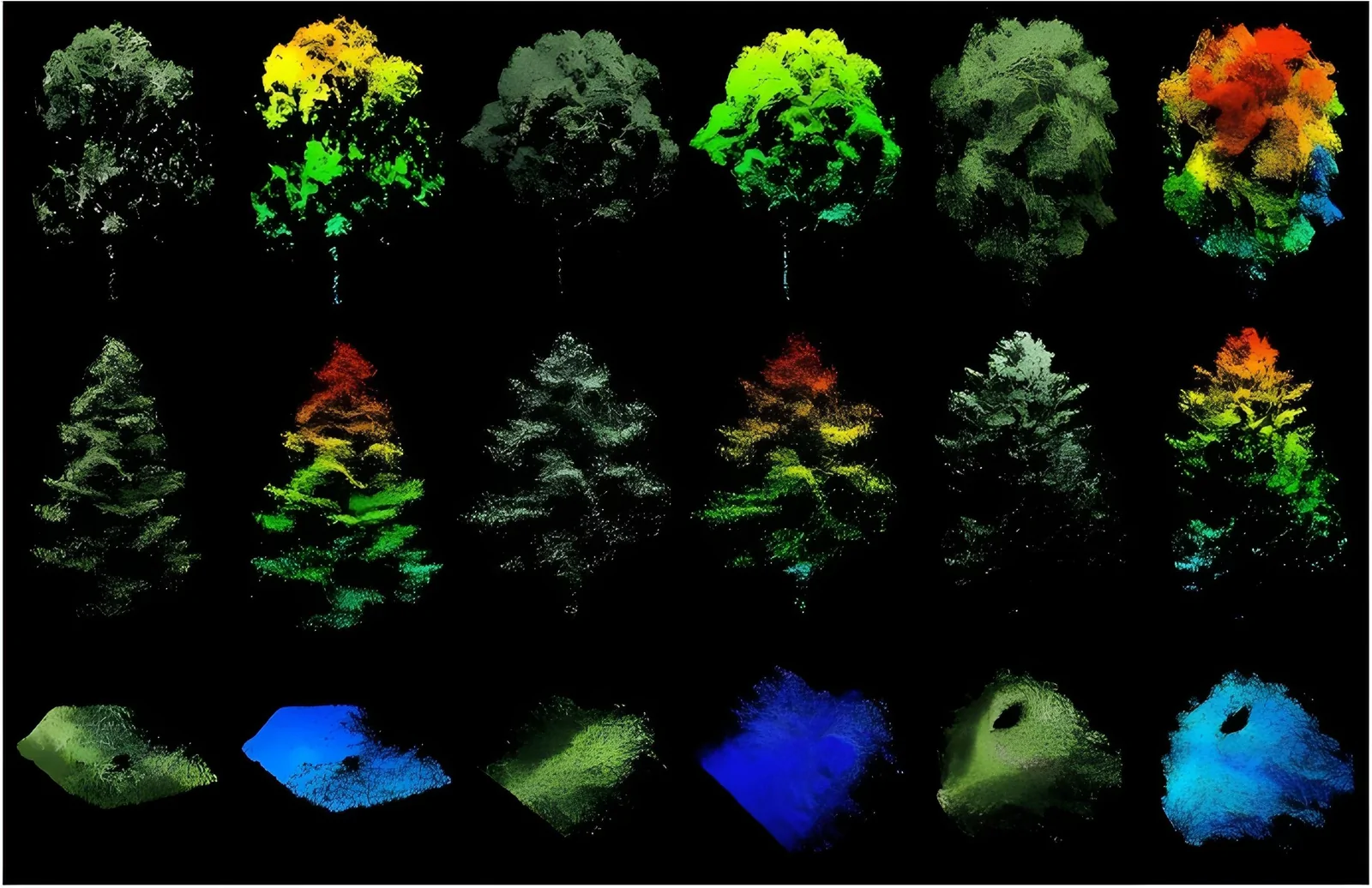
Point cloud data of broadleaf trees, coniferous trees and bare ground (shown with RGB and elevation).

To further evaluate the practical benefit of the proposed network, the original PointNet (Qi et al., 2017a) was added as a representative point-cloud deep learning baseline. PointNet was trained and tested under the same plot-level data split as PointNet++ and its variants. To ensure a fair comparison with the original PointNet++ baseline, PointNet used the same input features (the point coordinates X, Y, Z) and the same normalization to [−1,1]. The training settings were kept consistent with those used for PointNet++. After semantic segmentation, the subsequent tree localization and layer-stacking segmentation steps were kept unchanged.

#### 3.1.2 PointNet++ network architecture

PointNet++ is adopted as the backbone network for semantic segmentation because of its ability to capture local geometric structures from unordered point clouds. The network follows an encoder–decoder architecture. The encoder consists of multiple SA layers, each of which includes farthest point sampling, ball query grouping, and PointNet-based local feature extraction. The decoder uses feature propagation (FP) layers to upsample the learned features back to the original point resolution for per-point classification. To handle uneven point density, PointNet++ provides multi-scale grouping (MSG) and multi-resolution grouping (MRG) strategies. In this study, the MSG variant was selected.

Although PointNet++ can learn local structures effectively, the original network does not explicitly consider the importance and correlation of different input channels. In mixed coniferous and broadleaf forests, RGB, intensity, and echo-count channels provide complementary information that is critical for distinguishing tree species and ground points. Therefore, a channel attention mechanism and an Inverted Residual MLP (IRM) module were introduced to enhance the network’s feature extraction capability, as described in the following subsection.

#### 3.1.3 Channel attention mechanism

As the training set point cloud had multi-channel features, a channel attention mechanism was introduced into PointNet++ network to enhance the network’s feature extraction ability and reduce redundant information caused by channel expansion. The overall architecture of the PointNet++ network is illustrated in **Figure 3**. The channel attention mechanism constructed a mapping based on the relationship between channels. Then, this mapping was merged with the feature mapping through computation to obtain the feature mapping with enhanced channel attention (Jing et al., 2021). During the training process, each channel was assigned a weight value through an activation function, where higher weights indicated greater importance. To effectively capture the relationships between channels, the network’s feature representation was further improved by applying both max pooling and average pooling to aggregate channel features (Wang et al., 2022; P et al., 2021). The channel attention mechanism was embedded in each level of the SA layer, as shown in **Figure 4**.

**Figure 3:**
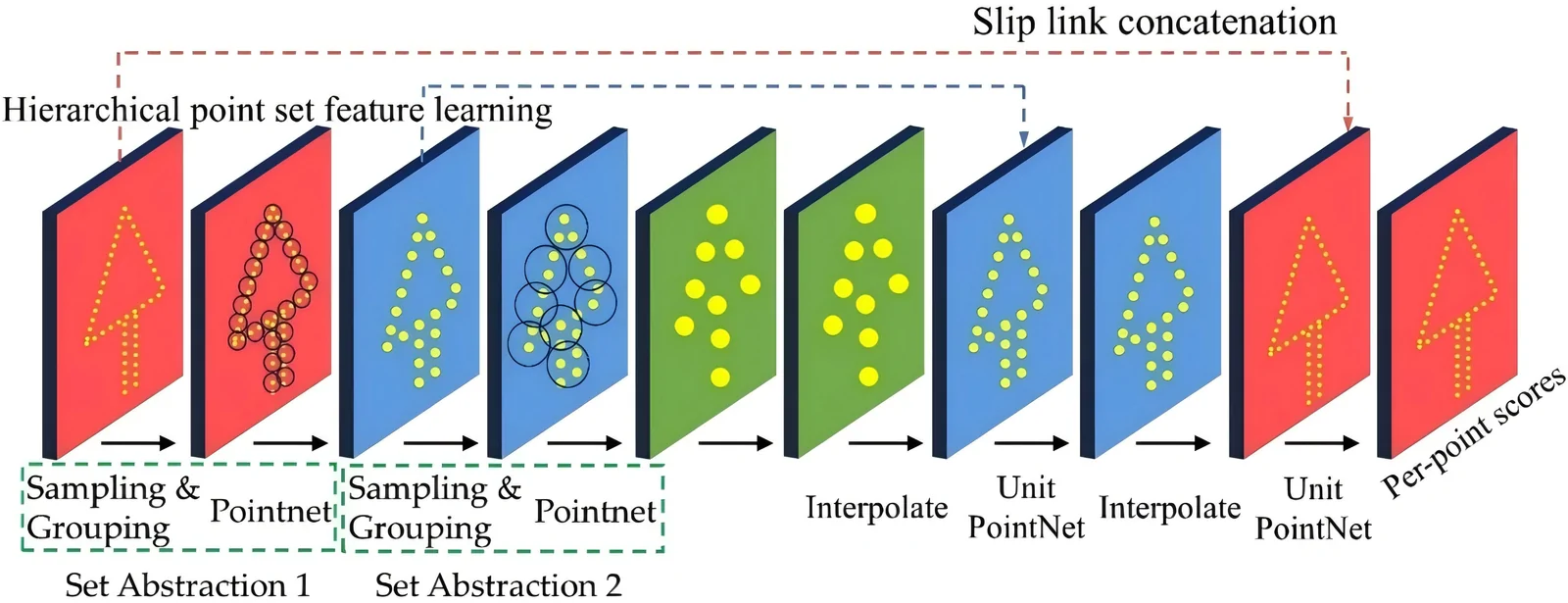
PointNet++ network architecture.

**Figure 4:**
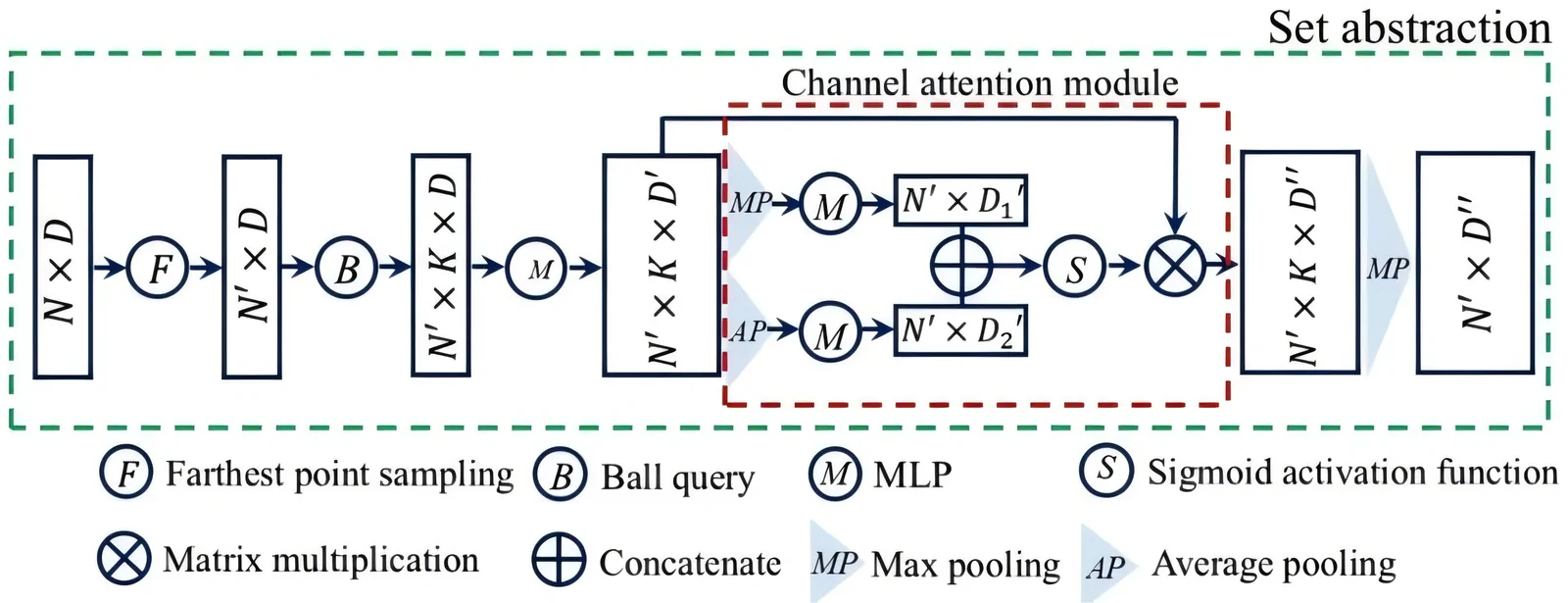
Network architecture of channel attention mechanism.

As shown in **Figure 4**, an N×D matrix was input at SA layer, where N represents the number of points and D represents the dimension of the feature vector. After farthest point sampling, a set of N’ points was obtained. In the grouping layer, the ball query algorithm was used to search for neighboring points around N’ centroids within a given radius. From these neighbors, K points were randomly selected to construct local regions, resulting in a point set of size N’×K×D. Each point’s features were extracted through a multi-layer perceptron (MLP) and output as a point set with a size of N’×K×D’. Then, maximum pooling and average pooling were separately applied to obtain the max-pooled feature with dimension D_1_’ and the average-pooled feature with dimension D_2_’. These two pooling features were processed separately through MLP layers and connected to obtain the fused feature with dimension D’’. Finally, the feature weight values were calculated using the sigmoid activation function and normalized to the range of [0,1]. After enhancing the important channels, the output matrix of size N’×D’’ was obtained through the pooling process again.

Additionally, to further enhance the performance of PointNet++ based on the channel attention mechanism, this study introduced the IRM module (Liu et al., 2025). In this module, the input features first passed through a 1x1 convolution layer for dimensionality reduction and then passed through the multi-layer MLP for multiple nonlinear transformations to capture deep features. Finally, the extracted features were upsampled and restored as the same dimension as the input features. In order to maintain the gradient fluidity of the network, skip connections were also included between the inverted residual blocks. The IRM module was designed to balance feature extraction capability and computational cost. However, the efficiency gain of IRM was not separately quantified in this study, and the accuracy improvement was mainly attributed to the combination of multi-feature input and the channel attention mechanism. The IRM module was placed after the SA layer, as shown in **Figure 5**.

**Figure 5:**
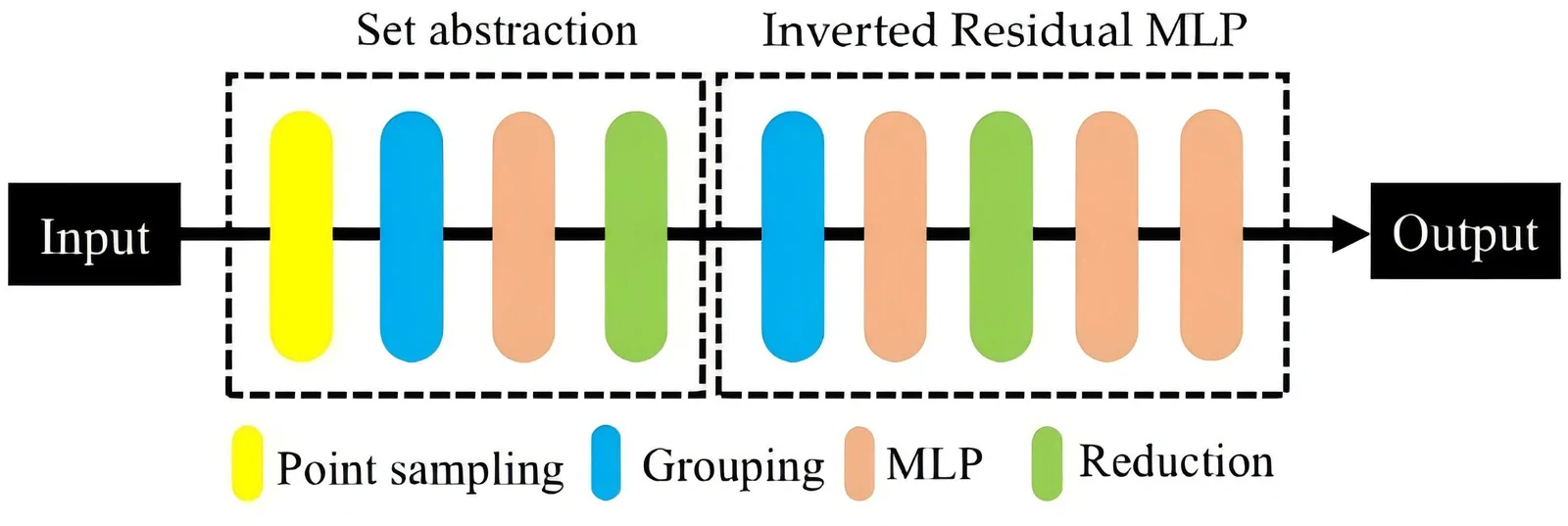
Network architecture of IRM.

In summary, the total training and testing time was approximately 2 hours. Detailed model settings are summarized in **Table 2**:

**Table 2:** Key model settings.

Item	Setting
Input points per sample	8192
Sampling method	Farthest point sampling (FPS)
SA layer 1	npoint=1024, radius=0.1, nsample=32, mlp=[32, 32, 64]
SA layer 2	npoint=256, radius=0.2, nsample=32, mlp=[64, 64, 128]
SA layer 3	npoint=64, radius=0.4, nsample=32, mlp=[128, 128, 256]
SA layer 4	npoint=16, radius=0.8, nsample=32, mlp=[256, 256, 512]
Optimizer	Adam
Initial learning rate	0.001
Learning-rate schedule	Step decay (halved every 20 epochs)
Loss function	Cross-entropy loss
Batch size	16
Training epochs	250
Data augmentation	Random scaling and translation
Model selection	Fixed training epochs
Hardware	NVIDIA Quadro RTX 3000
Software	Python 3.7, TensorFlow 1.13, CUDA 10.0, cuDNN 7.4

The input features consist of point coordinates (X, Y, Z), RGB values (R, G, B), laser intensity (I), and echo count (N). Point coordinates are normalized to [−1, 1] before being fed into the network.

#### 3.1.4 Individual tree point clouds extraction

The PointNet++ network was used only for semantic segmentation, which classified the point cloud into coniferous trees, broadleaf trees, and ground points. The extraction of individual trees was achieved by a subsequent pipeline consisting of tree localization and layer-stacking segmentation. After removing the ground points, a method that combined local highest point and density maximum point for individual tree segmentation was proposed. The specific steps were as follows:

1. Tree top was the highest point within the crown area. First, the tree point clouds were projected onto the XOZ plane. Then, the local maximum value filtering with a variable window was used to find the position of the tree top, which was the point with the maximum Z value within the neighborhood (Pan., 2022). Therefore, these points need to be filtered and removed before extracting the maximum value points. For two points Pi(xi,yi,zi) and Pj(xj,yj,zj)(xi=xj; i=1,2,3…; j=1,2,3…), if zi>zj, point Pj was removed; if zi<zj, point Pi was removed; when zi=zj, any one of the points was kept. The above steps were repeated and the remaining point set was represented as T={T(xi,yi,zi)/i=1,2,3…n}. For any point T’(xj,yj,zj) in the set T, if zj>zj+1 and zj<zj-1, the point was considered as a local highest point within the neighborhood, which was the tree top.
2. Due to the presence of crowns deviation and multiple crown tops, local tree tops cannot fully represent the actual tree location (Liu et al., 2022). Based on the characteristics of UAV LiDAR scanning, areas with higher point cloud density indicated more leaves and branches, which were also closer to the center of the tree. Therefore, this study proposed a secondary positioning method based on density maximum points for tree location. After projecting the tree point clouds onto the XOY plane, the Hough transform was used to automatically detect tree crown size. Based on the detection results, the tree crowns of coniferous and broadleaf trees were separately divided into grids. Each detected tree crown circle was ensured to be covered by a 3×3 grid, as shown in **Figure 5C**. The number of points N in each grid was counted and divided by the grid area S to obtain the grid density d. By comparing the densities d within the neighborhood, the maximum value d’ was obtained. The center point of the grid d’ was considered as a density extremum point, and the set of these points was represented as D={D(xi,yi,zi)/i=1,2,3…n}.
3. The threshold a is referred to as the fusion parameter in this study. It represents the distance threshold used to combine local highest points and density maximum points. For a given fusion parameter a, the distance d was calculated between any local highest point Ti(xi,yi,zi) and all density maximum points Dj(xj,yj,zj). If d<a, the center point of the line connecting Ti and Dj was taken as the tree location point and deleted both Ti and Dj; if d>a, the Ti was directly deleted. The unit of a is meters. The values a=1 for coniferous trees and a=2 for broadleaf trees were determined on the training plots based on the differences in crown size and point cloud density between the two tree types. Broadleaf trees generally have larger and more irregular crowns, so a larger fusion distance was used. These values were fixed before evaluating the independent test plots. The above steps were repeated until all maximum points in the set T were traversed. The obtained tree location points and the remaining points were combined and represented as set L={Li/i=1,2,3…n}, and this point set was considered as the final tree location points. Finally, individual tree segmentation was achieved based on the extracted tree location points using layer-stacking segmentation algorithm (Liu et al., 2023). **Figure 6** shows the process of determining tree location points through the example point cloud of trees:

**Figure 6:**
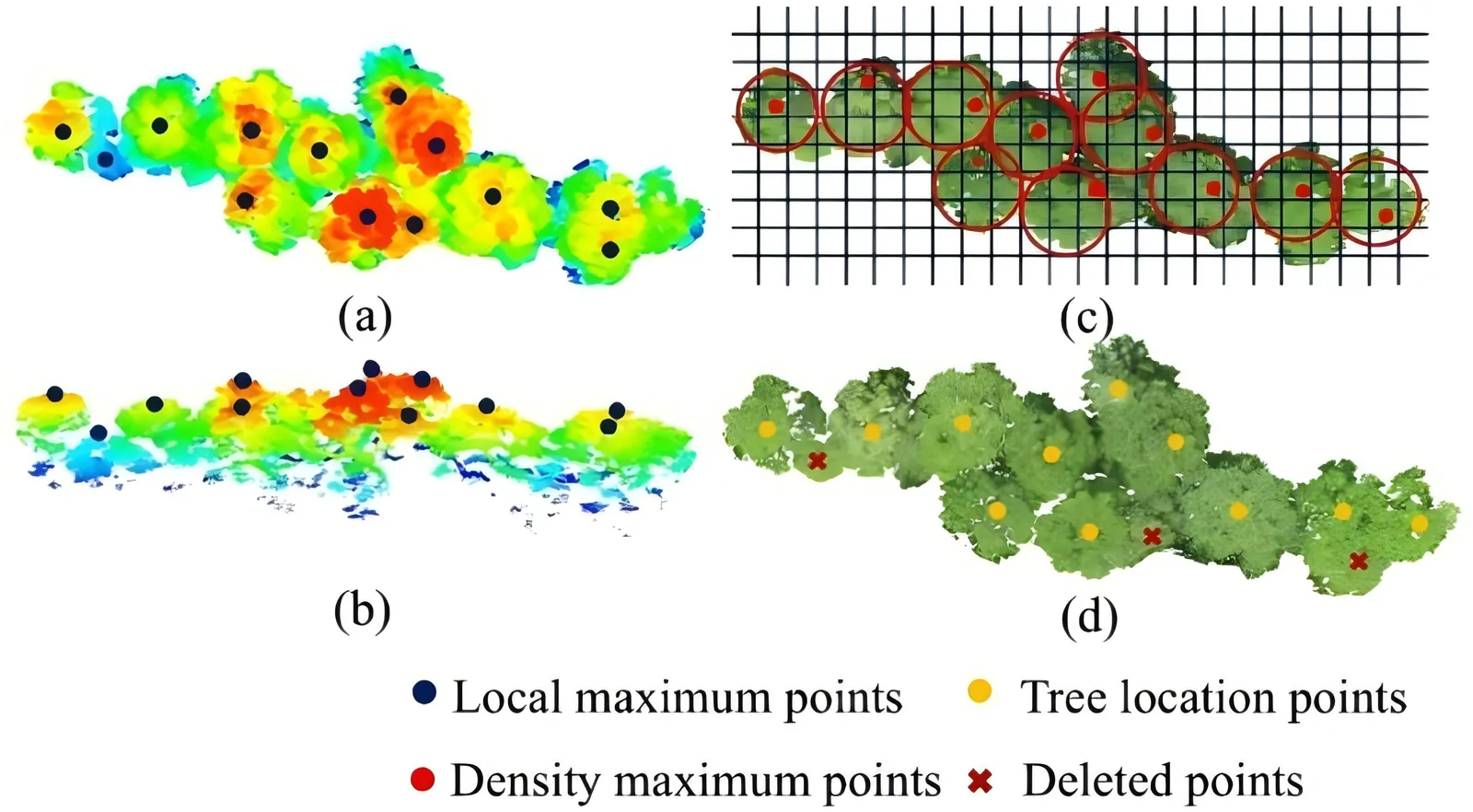
The process of determining the location of trees. **(a, b)** show the determination of local highest points by Z value information (top view and side view); **(c)** show the determination of density maximum points by grid method; **(d)** show the determination of tree location points.

### 3.2 Other individual tree segmentation algorithms

#### 3.2.1 Marker-based watershed algorithm

The marker-based watershed algorithm is a morphological segmentation method based on topological theory (Chen et al., 2006). When applied to tree point clouds segmentation, the point cloud data need to be converted into a Canopy Height Model (CHM). The CHM represents the height of objects on the ground, such as vegetation and buildings, which is obtained by subtracting the Digital Surface Model (DSM) from the Digital Elevation Model (DEM). Generally, the DEM is generated by irregular triangulation, while the DSM is generated using inverse distance weighting interpolation. Finally, CHMs with different spatial resolutions (0.1 m, 0.3 m, 0.5 m, and 1.0 m) were used for crown segmentation, allowing for the identification of individual tree locations and boundaries. The CHM resolution was set to 0.5 m based on previous studies (Yan et al., 2026). This parameter was fixed before evaluating the test plots and was not tuned on the test data.

In the marker-based watershed algorithm, local maximum points in the CHM are considered as peaks and the local minimum points are considered as valleys. This algorithm simulates the process of water flowing into valleys to determine tree top positions and crown boundaries. However, the traditional watershed algorithm is often sensitive to noise, which can easily lead to over-segmentation. Therefore, the marker-based watershed algorithm searches for the potential tree vertices by introducing local maximum filtering, and then optimizes the segmentation process.

#### 3.2.2 Distance-based segmentation algorithm

The distance-based segmentation algorithm (Li et al., 2012) classifies points based on the distance between adjacent trees. It processes points sequentially from the highest to the lowest point of the tree, and achieves individual tree segmentation through iterative calculations. The algorithm starts with the global maximum point as the initial seed point. Points farther than a specified threshold from the seed are excluded, while those within the threshold are classified using the minimum distance rule, as illustrated in **Figure 7**. Point A is the global highest point, it is considered as the tree top point of Tree 1. Points B, C, and D are subsequently classified. Firstly, since the horizontal distance d_AB_ between point B and point A is greater than the set threshold, point B is assigned as a point of Tree 2. On the other hand, the horizontal distance d_AC_ between point C and point A is smaller than the set threshold, and d_AC_ is smaller than d_BC_, so point C is assigned as a point of Tree 1. Similarly, since d_BD_ is smaller than d_CD_, point D is assigned as a point of Tree 2. By defining an appropriate distance threshold, most points can be correctly assigned to corresponding tree clusters. The threshold should be approximately equal to the crown radius. If the threshold is too small, trees with elongated branches may be overly segmented. If the threshold is too large, adjacent trees may be incorrectly segmented as the same tree. The distance threshold was set to 2 m based on the tree spacing in the training plots. This parameter was fixed before evaluating the test plots and was not tuned on the test data.

**Figure 7:**
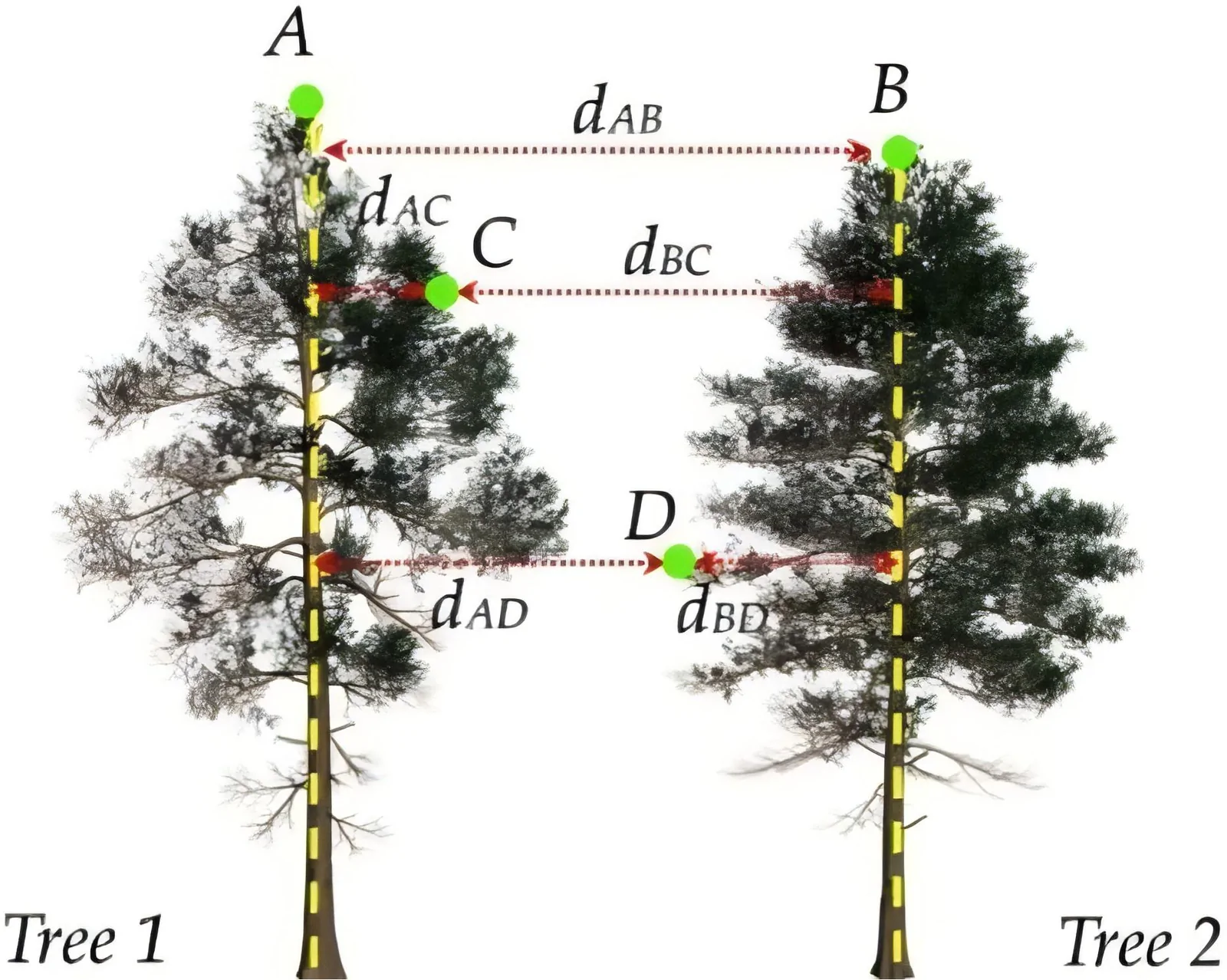
Schematic diagram of individual tree segmentation based on distance-based segmentation algorithm.

#### 3.2.3 Mean shift algorithm

Mean shift algorithm is a point-based clustering algorithm that identifies the densest regions in a point cloud through iterative updates. The process begins with selecting a random point as the initial center. Firstly, a random point is chosen as the initial point, and the mean offset of all points within the bandwidth is calculated. Then, the initial point is moved to the location of the mean offset and becomes the new starting point. This process is iteratively repeated until the final condition is met. To address the difficulty of determining the bandwidth parameter in mixed forest with different crown sizes, this study adopted the adaptive bandwidth Mean shift algorithm proposed by Yan et al. (2020). This algorithm can automatically estimate the optimal kernel bandwidth based on the sample crown size without prior knowledge. Firstly, the global maximum point is taken as the initial center, and the 3D space is divided into a set of angular sectors. For different angular sectors, potential tree surface points are extracted using the vertical profiles of the point cloud. Then, based on the simulated tree crown surface and considering the influence of tree spacing, the optimal kernel bandwidth value is automatically estimated. Iterative calculation of this method within the given area, individual tree points are divided by continuously shifting the clustering center until all trees are segmented. The method was applied with its default settings in this study.

### 3.3 Accuracy assessment

#### 3.3.1 Evaluation of semantic segmentation results

Intersection over Union (IoU) is used for each class to evaluate the effectiveness of semantic segmentation. Mean Intersection over Union (mIoU) is the average IoU across all classes. Overall Accuracy (OA) is the ratio of correctly classified samples to the total number of samples (Gao and Zhang, 2022). The formulas for calculating IoU, mIoU, and OA are given by Equations 1–3 respectively.

(1) $$IoU=\frac{tp}{fn+fp+tp}$$

(2) $$mIoU=\frac{1}{k+1}\sum_{i=0}^{k}\frac{tp}{fn+fp+tp}$$

(3) $$OA=\frac{tp+tn}{fn+fp+tp+tn}$$

Here, tp represents the true positive points; fp represents the false positive points; fn represents the false negative points; tn represents the true negative points.

#### 3.3.2 Evaluation of individual tree segmentation

The accuracy of tree localization is an important criterion for individual tree segmentation. The degree of deviation was introduced to evaluate the extraction results of tree location points in this study. Based on field measurements, the average crown width of trees within the sample plots was 8.9m, and the maximum crown width was 15.6m. Therefore, constructing a circle with a radius of 10m centered on the actual location of the tree was more conducive to the calculation of deviation. This 10 m value was therefore defined as the matching threshold. For tree-level accuracy assessment, each reference tree was matched to the nearest extracted tree within the 10 m threshold. If multiple extracted trees fell within the threshold, the closest one was chosen. The degree of deviation was defined as the ratio of the distance between the actual tree location points and the extracted tree location points to the radius. tree location points beyond the 10 m range were discarded.tree location. A higher degree of deviation indicated a greater distance between the extracted tree location points and the actual tree location points. The calculation equation is given in Equation 4:

(4) $$D=\frac{d}{R}\times 100\%$$

where, d represents the distance between the extracted tree location points and the actual tree location points; R represents the radius of the circle.

The performance of individual tree segmentation is evaluated by three indices: precision rate (p), recall rate (r), and F-score (F1) (Parkan and Tuia, 2018), as shown in Equations 5–7. Where, p represents the proportion of correctly segmented individual trees by the algorithm, with a value closer to 1 indicating more accurate segmentation. The r represents the proportion of correctly segmented individual trees to the actual number of trees in the plot, with a value closer to 1 indicating more individual trees correctly segmented and better segmentation performance. F1 is a measure that overall evaluates the segmentation quality, with a value closer to 1 indicating better overall performance.

(5) $$p=\frac{TP}{TP+FP}$$

(6) $$r=\frac{TP}{TP+FN}$$

(7) $$F1=\frac{2\times r\times p}{r+p}$$

Here, TP represents the number of correctly segmented trees; FN represents the number of missed trees; FP represents the number of falsely detected trees. Moreover, the matching threshold for precision, recall, and F1 was set to 10 m, identical to the radius used for the positional deviation assessment.

#### 3.3.3 Evaluation of individual tree point clouds segmentation quality

After segmentation, the quality of point clouds among individual trees often varies. To further evaluate the segmentation quality, the integrity of individual tree point clouds was introduced in this study (Liu et al., 2018, 2022). It referred to the proportion of tree points to the actual tree points when a tree was separated from the plots. The actual tree points were obtained through manual visual interpretation. Based on the integrity of individual tree point clouds, the Equations 8–10 can be derived as follows:

(8) $$N_{TP}=\frac{n_{1}}{n}\times 100\%$$

(9) $$N_{FN}=\frac{\left(n_{-}n_{2}\right)}{n}\times 100\%$$

(10) $$N_{FP}=\frac{\left(n_{1}-n_{2}\right)}{n}\times 100\%$$

where, N_TP_ represents the proportion of correctly segmented points in the individual tree point clouds; N_FN_ represents the proportion of under-segmented points in the individual tree point clouds; N_FP_ represents the proportion of over-segmented points in the individual tree point clouds; n represents actual number of individual tree points; n_1_ represents the extracted number of individual tree points; n_2_ represents the number of points in the overlapping region between the actual and the extracted individual tree.

## 4 Results

### 4.1 Results of individual tree segmentation based on PointNet++ network

The semantic segmentation metrics and individual tree segmentation evaluation metrics reported in this study were the mean values across 5 repeated training runs. The same plot-level data split was used in all runs, and the five test plots were kept independent from training and parameter selection. These repeated runs were performed to ensure stable average performance.

#### 4.1.1 Semantic segmentation analysis

**Figure 8** shows the change in loss and accuracy for the original PointNet++ network and the PointNet++ network with channel attention mechanism. In the training and testing accuracy curves, the growth rate of the original PointNet++ network stabilized after epoch=156. The PointNet++ network with multi-feature input achieved approximately 3% higher accuracy than the original network. However, its accuracy curve did not stabilize until epoch=189. This indicated that while incorporating multi-feature information improved accuracy, it also slowed down the network’s convergence speed. The channel attention mechanism and IRM module were introduced to improve feature representation and training convergence. The improved PointNet++ reached stable accuracy in fewer epochs than the multi-feature PointNet++ without these modules, and the optimal accuracy was improved by approximately 2%.

**Figure 8:**
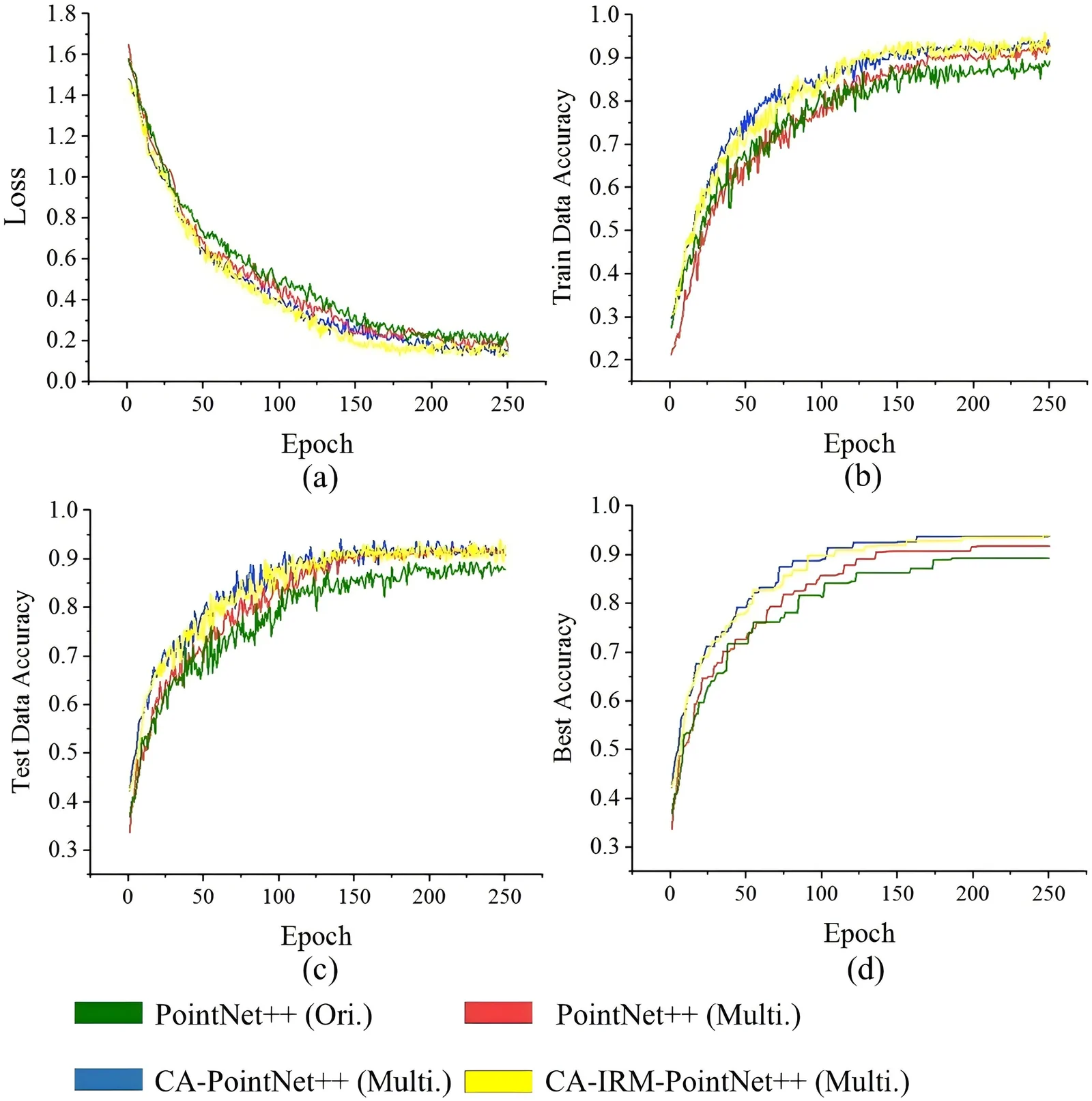
Changes in the loss function and accuracy during model training. **(a)** shows the curve of the loss function; **(b)** shows the curve of the train data accuracy; **(c)** shows the curve of the test data accuracy; **(d)** shows the curve of the best accuracy. PointNet++ (Ori.): Original PointNet++ adopted standard data format (X, Y, Z); PointNet++ (Multi.): Multi-characteristic PointNet++ adopted data format of (X, Y, Z; R, G, B; I; N); CA-PointNet++ (Multi.): PointNet++ improved by channel attention; CA-IRM-PointNet++ (Multi.): PointNet++ improved by channel attention and IRM.

Two semantic segmentation test sets were selected as input data for both the original PointNet++ network and the PointNet++ network with channel attention mechanism. One test set contained only XYZ coordinate information, while the other test set contained multiple feature information (X, Y, Z; R, G, B; I; N). The semantic segmentation results reported in **Table 3** were also obtained from the five test plots. The semantic segmentation results for the plots were shown in **Figure 9**. As shown in **Table 3**, PointNet achieved an OA of 78.96% and an mIoU of 70.14%, which were lower than those of PointNet++ (Ori.) and its variants. This likely occurs because PointNet relies primarily on global max pooling for feature aggregation and lacks hierarchical local feature learning, thereby reducing its ability to differentiate coniferous from broadleaf trees under overlapping crowns and irregular canopy structures. In contrast, PointNet++ introduces set abstraction layers to capture local geometric structures at multiple scales. In addition, the statistical results showed that applying multi-feature data to the semantic segmentation network can improve the segmentation accuracy of each category to varying degrees. Compared to the PointNet++ network with only XYZ coordinate information, the overall accuracy (OA) improved by 3.06% and the mIoU improved by 5.64%. In different categories, it was evident that the introduction of multi-feature information significantly improved the IoU of coniferous and broadleaf trees. The average IoU of tree point clouds and ground points increased by 7.13% and 2.66%, respectively. Additionally, with the inclusion of the channel attention mechanism and IRM module, the OA and mIoU obtained from the semantic segmentation results further improved (OA = 93.91%, mIoU = 90.49%). Among all categories, coniferous trees showed the largest improvement in IoU, approximately 4.53%. Therefore, the inclusion of local information from point clouds and the channel attention mechanism effectively enhanced the semantic segmentation capability of the network. On the other hand, due to the significantly larger volume of broadleaf trees compared to coniferous trees and the patchy distribution of coniferous trees, the recognition ability of the PointNet++ deep learning algorithm for broadleaf tree point clouds was slightly higher than that for coniferous trees. The ground points achieved the highest semantic segmentation accuracy, which can be attributed to its prominent height features.

**Table 3:** Statistical results of overall accuracy (OA) and intersection over union (IoU) across coniferous trees, broadleaf trees, and ground points on the five test plots after semantic segmentation using PointNet, PointNet++, and PointNet++ variants under the same data split.

Methods	OA	IoU-Coniferous trees	IoU-Broadleaf trees	IoU-Ground	mIoU
PointNet (Ori.)	78.96%	62.53%	68.85%	79.04%	70.14%
PointNet++ (Ori.)	88.67%	75.24%	80.49%	88.81%	81.51%
PointNet++ (Multi.)	91.73%	83.22%	86.76%	91.47%	87.15%
CA-PointNet++(Multi.)	93.82%	87.69%	90.13%	93.26%	90.36%
CA-IRM-PointNet++(Multi.)	93.91%	87.75%	90.35%	93.38%	90.49%

Values are means across 5 repeated training runs.

**Figure 9:**
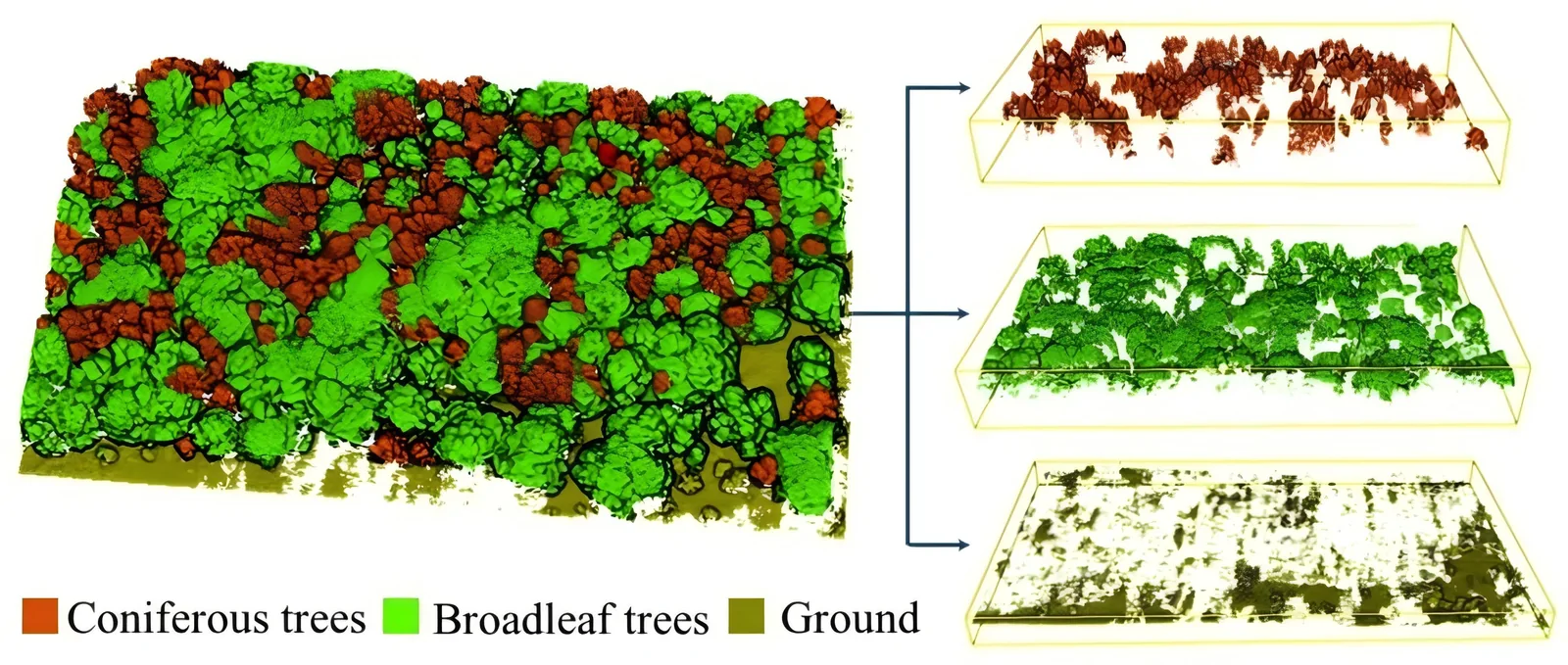
Results of semantic segmentation for the testing plot by CA-IRM-PointNet++ based on test plots.

#### 4.1.2 Analysis of individual trees extraction results

After performing semantic segmentation of tree point clouds using the PointNet++ network, three localization strategies were compared in this study: (1) using only local highest points as tree locations, (2) using only density maximum points as tree locations, and (3) the proposed combination of local highest points and density maximum points. For strategies (1) and (2), the same layer-stacking segmentation algorithm was applied to the extracted tree location points. For strategy (3), the fusion rule described in Section 3.1 was used. All other parameters were kept identical across the three strategies tree location. To focus on the overall contribution of the localization step, the comparison of localization strategies is reported for all trees (**Table 4**). As shown in **Table 4**, using only local highest points or only density maximum points resulted in lower tree-level F1 scores (0.790 and 0.823, respectively) than the proposed combination (0.843). This indicates that both local highest points and density maximum points provide complementary information for tree localization, and the downstream localization step contributes to the final individual tree segmentation accuracy. Furthermore, the accuracy of tree location points extraction was evaluated based on the degree of deviation, as shown in **Figure 10**. Compared to the density maximum points, local highest points exhibited a greater deviation from the actual location of trees, with about 1/3 of the points deviating by more than 50%. In the process of detecting tree tops, the local maximum method cannot accurately determine the actual tree location, especially in broadleaf forest. Therefore, local highest points cannot be used to replace the tree location points in mixed coniferous and broadleaf forest. Additionally, the deviation from actual tree locations was effectively reduced by combining local highest points and density maximum points to obtain matching points, and the degree of deviations were mostly between 10%-30%. On the other hand, there were also significant differences in the extraction results of tree locations based on coniferous and broadleaf trees. For coniferous trees, over 90% of extracted locations had deviations within 50%.; In contrast, the deviation of tree location extraction was widely distributed between 20% and 70% for broadleaf trees, which indicated that the extraction error was more discrete. Based on the extracted tree location points, individual tree segmentation was achieved using the layer-stacking segmentation algorithm, and the results are shown in **Figure 11**. From **Figure 11**, the proposed algorithm demonstrated good segmentation effects for both coniferous and broadleaf trees in the sample plots.

**Table 4:** Comparison of tree localization strategies under fixed semantic segmentation and layer-stacking segmentation based on CA-IRM-PointNet++ (recall *r*, precision *p*, and F1-score *F*1).

Localization strategy	*r*	*p*	*F*1
Local highest points only	0.782	0.798	0.790
Density maximum points only	0.820	0.827	0.823
Combined(proposed)	0.836	0.849	0.843

**Figure 10:**
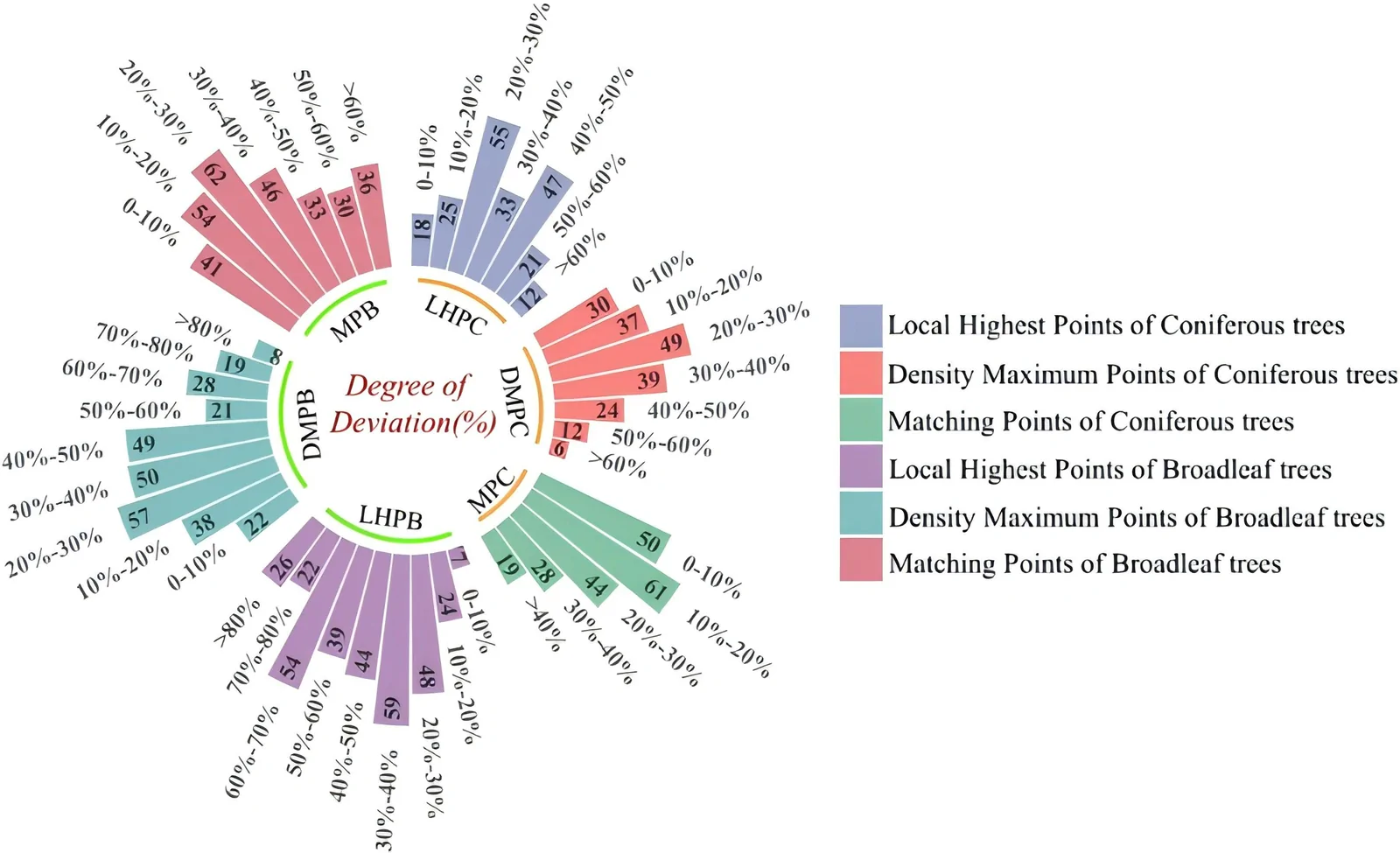
The statistics of deviation from actual tree location.

**Figure 11:**
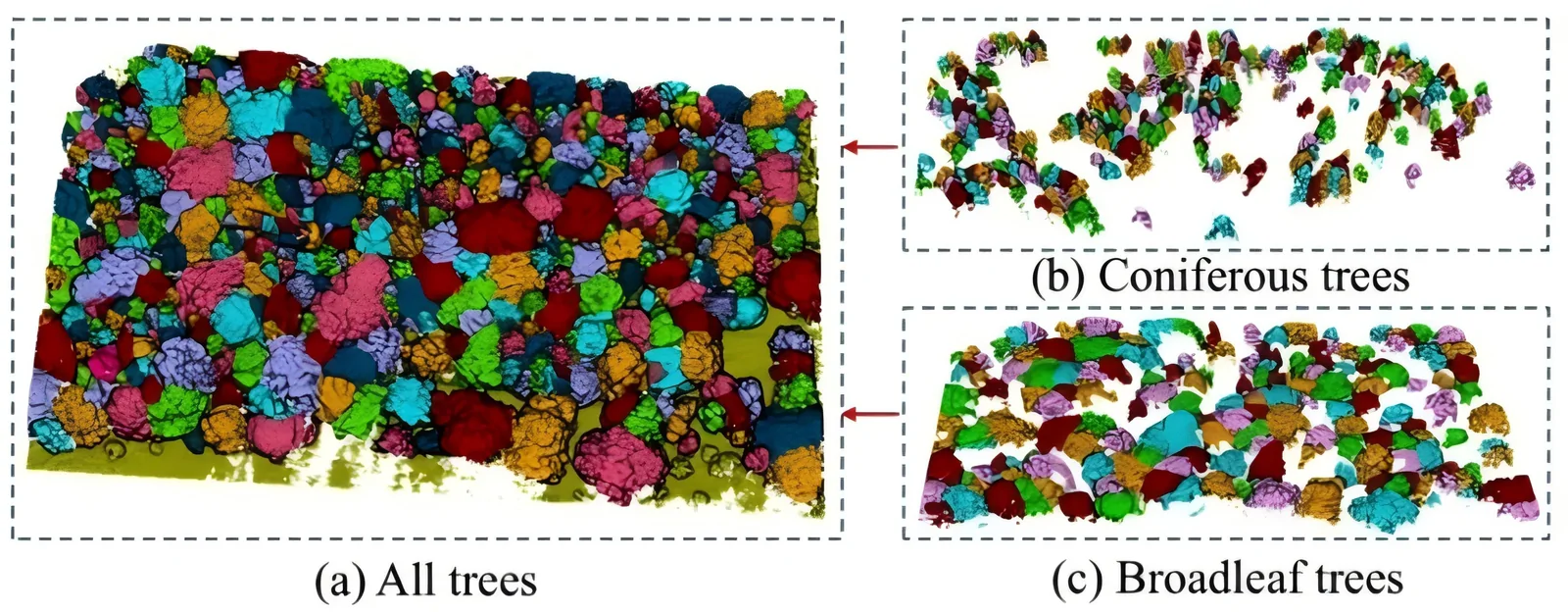
Results of individual tree segmentation based on test plots.

### 4.2 Comparative analysis of segmentation results

The proposed method in this study was compared with the marker-based watershed algorithm, distance-based segmentation algorithm, mean shift algorithm and PointNet. All algorithms were evaluated on the same independent test plots to ensure a fair comparison. **Table 5** quantitatively summarizes the segmentation accuracy of different segmentation methods on the five test plots. Although the marker-based watershed algorithm was simple and easy to implement, it tended to suffer from over-segmentation issues due to the presence of significant tree canopy occlusion in mixed coniferous and broadleaf forest. Both the distance-based segmentation algorithm and Mean shift algorithm showed improved accuracy compared to the marker-based watershed algorithm. However, there was uncertainty in parameter settings and initial clustering determination in the mixed forest, which may affect the segmentation accuracy. In contrast, the PointNet++-based segmentation method improved accuracy by approximately 6.5% to 20.5% compared to these three methods. This result was mainly due to the precise separation of tree point clouds and accurate determination of tree locations. Moreover, PointNet achieved an F1 of 0.827 for all trees, 0.840 for coniferous trees, and 0.807 for broadleaf trees, which were lower than those of the proposed CA-IRM-PointNet++ (F1 = 0.843, 0.865, and 0.828, respectively). To assess the consistency of the proposed CA-IRM-PointNet++ across different test plots, the tree-level F1 was calculated separately for each of the five independent held-out test plots. The F1 scores ranged from 0.819 to 0.860 across the five plots, with no plot showing a substantial deviation from the overall result. This indicates that the proposed CA-IRM-PointNet++ performed relatively consistently across the studied forest conditions. From the segmentation accuracy of different types of trees, coniferous trees generally performed better than broadleaf trees. Traditional algorithms exhibited a large number of over-segmentation occurrences in the segmentation of broadleaf trees. Due to the difficulty in coordinating the setting of global and local optimal parameters, many broadleaf trees in mixed forest were mistakenly identified as combinations of multiple coniferous trees. This issue was particularly evident in the marker-based watershed algorithm, with the p value of only 0.716. The improved PointNet++ algorithm utilized semantic segmentation to mitigate the parameter adjustment challenges caused by mixed coniferous and broadleaf forest, reducing the complexity of subsequent instance segmentation. The results indicated that this method achieved optimal accuracy for both coniferous and broadleaf tree segmentation, with scores of 0.859 and 0.821, respectively. Notably, the segmentation accuracy for broadleaf trees remained slightly lower than that of coniferous trees. This suggested that broadleaf tree point clouds exhibited lower stability during segmentation and were more susceptible to external disturbances.

**Table 5:** Comparison of individual tree segmentation accuracy (recall *r*, precision *p*, and F1-score *F*1) among four algorithms (marker-based watershed, distance-based segmentation, mean shift, PointNet and the proposed CA-IRM-PointNet++) on the five independent test plots in mixed coniferous and broadleaf forest.

Types	Algorithms	*r*	*p*	*F*1
All trees	Marker-based Watershed	0.631	0.716	0.671
	Distance-based Segmentation	0.721	0.774	0.746
	Mean shift	0.771	0.793	0.782
	PointNet	0.820	0.835	0.827
	CA-IRM-PointNet++	0.836	0.849	0.843
Coniferous trees	Marker-based Watershed	0.629	0.741	0.681
	Distance-based Segmentation	0.742	0.784	0.762
	Mean shift	0.785	0.809	0.797
	PointNet	0.833	0.848	0.840
	CA-IRM-PointNet++	0.859	0.871	0.865
Broadleaf trees	Marker-based Watershed	0.632	0.700	0.664
	Distance-based Segmentation	0.707	0.767	0.736
	Mean shift	0.762	0.783	0.772
	PointNet	0.798	0.817	0.807
	CA-IRM-PointNet++	0.821	0.834	0.828

Values are means across 5 repeated training runs.

### 4.3 Sensitivity analysis of matching threshold and fusion parameters

To evaluate the dependence of the proposed method on key parameter choices, a sensitivity analysis was performed on the training plots using the proposed CA-IRM-PointNet++ model. The matching threshold and the fusion parameter a were varied while keeping the semantic segmentation results and the layer-stacking segmentation unchanged. The parameters used in the main experiment were determined based on field-measured crown widths and preliminary tests on the training plots. The sensitivity analysis was therefore performed only on the training plots to avoid parameter tuning on the final test data.

For the matching threshold, values from 5 m to 15 m were tested. As shown in **Table 6**, the tree-level F1 remained relatively stable across the tested range. The variation in F1 was small, indicating that the evaluation is not highly sensitive to the matching threshold. For the fusion parameter a, values from 0.5 to 3.0 were tested for coniferous and broadleaf trees. As shown in **Table 7**, the tree-level F1 changed only slightly across the tested range. The highest F1 was obtained at a=1 for coniferous trees and a=2 for broadleaf trees, which is consistent with the parameter values used in this study.

**Table 6:** Sensitivity of tree-level F1 to the matching threshold on the training plots.

Matching threshold (m)	*F*1 (All trees)
5.0	0.821
7.5	0.832
10.0	0.837
12.5	0.830
15.0	0.825

**Table 7:** Sensitivity of tree-level F1 to the fusion parameter *a* on the training plots.

Fusion parameter *a* (m)	*F*1 (Coniferous trees)	*F*1 (Broadleaf trees)
0.5	0.845	0.798
1.0	0.851	0.805
1.5	0.847	0.813
2.0	0.842	0.819
2.5	0.834	0.816
3.0	0.823	0.808

Overall, the sensitivity analysis confirms that the proposed localization and segmentation pipeline is relatively robust to the matching threshold and the fusion parameter. The final values were determined on the training plots and fixed for the independent test plots, ensuring that parameter selection remained independent of the final evaluation data.

### 4.4 Classification results of tree types

The visual analysis of segmented trees from the sample plots revealed significant differences in point cloud segmentation quality among individual trees, as shown in **Figure 12**. In coniferous trees, most overlapping crown areas appeared in the middle and lower layers. In contrast, broadleaf trees showed varying degrees of crown overlap in the middle and upper layers. Therefore, the integrity was introduced to evaluate the segmentation quality of individual tree point clouds. Based on the different proportions of points with correct segmentation, under-segmentation and over-segmentation, the number distribution of coniferous and broadleaf trees was statistically analyzed by heat map, as shown in **Figure 13**. Specifically, the horizontal axis represented the four segmentation algorithms and the two tree categories; The vertical axis represented the proportion of points with correct segmentation, under-segmentation and over-segmentation within individual tree point clouds; The color variations in the heatmap reflected differences in tree quantity. From the influence of different algorithms, the NFN obtained by the marker-based watershed algorithm was concentrated between 50% and 70%, and the NFP was concentrated between 30% and 40%. This indicated a large number of under-segmented point clouds during the tree segmentation process. The point cloud quality of coniferous and broadleaf trees segmented using the point cloud segmentation (PCS) algorithm showed significant differences. Conversely, the Mean shift algorithm produced a more balanced point cloud quality for both tree types. The algorithm based on the PointNet++ achieved the highest quality of individual tree point clouds segmentation (mean NTP = 87.9%), and the NFN was mostly concentrated around 10%, and the NFP was concentrated between 5% and 10%, which indicated that the algorithm had better robustness in the process of segmentation. For different forest types, The NTP of coniferous tree point clouds was generally more than 70%, indicating a low proportion of misclassified points and high-quality individual tree segmentation. In contrast, approximately 1/3 of broadleaf tree point clouds had the value of NTP below 50%, and values of NFN and NFP were mostly above 30% for these lower-quality segmentations. This indicated that broadleaf point cloud had a higher proportion of segmentation errors compared to coniferous trees, and its segmentation results were more affected by the canopy overlap.

**Figure 12:**
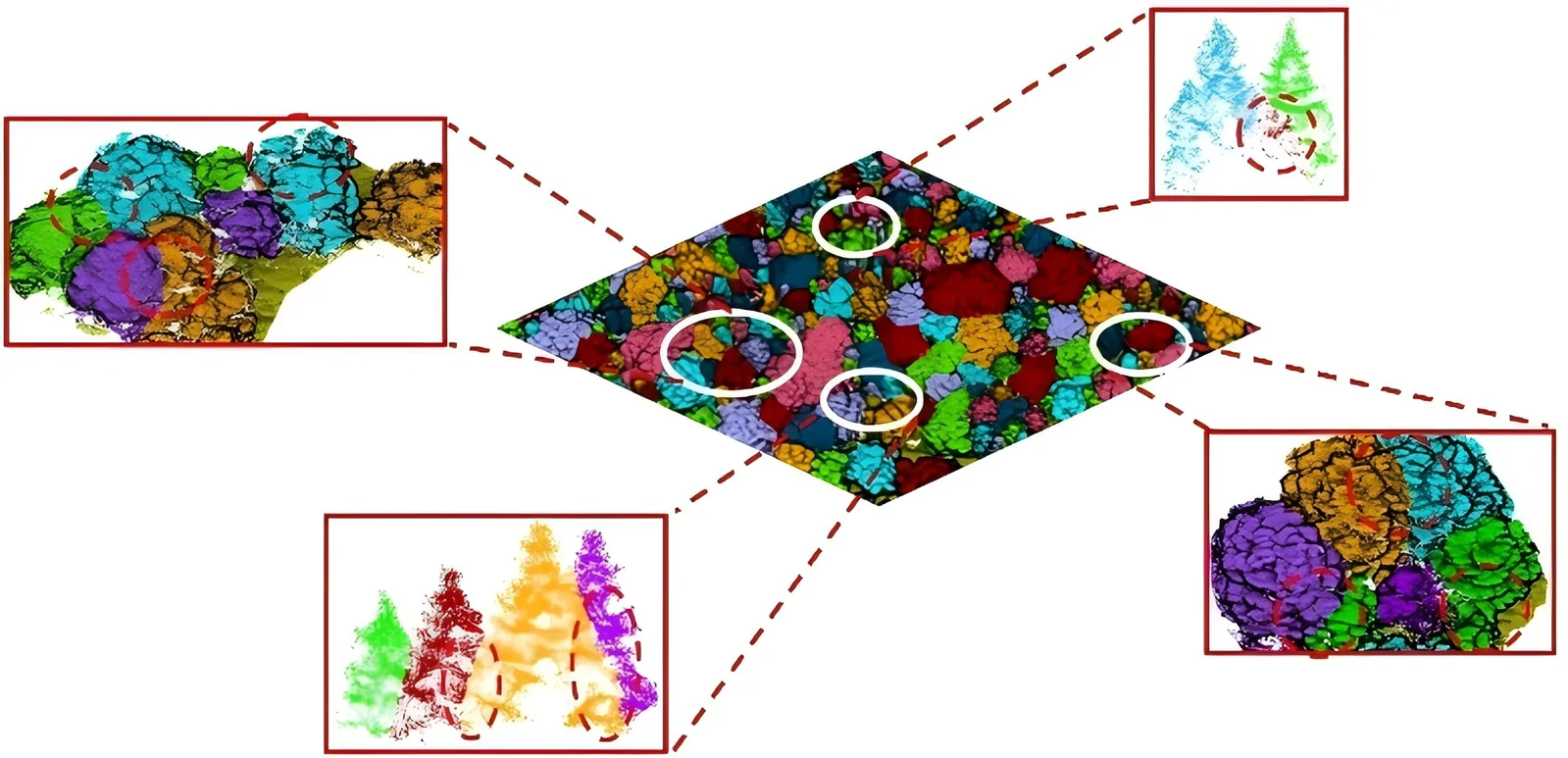
Examples of mis-segmented individual tree point clouds in test plots.

**Figure 13:**
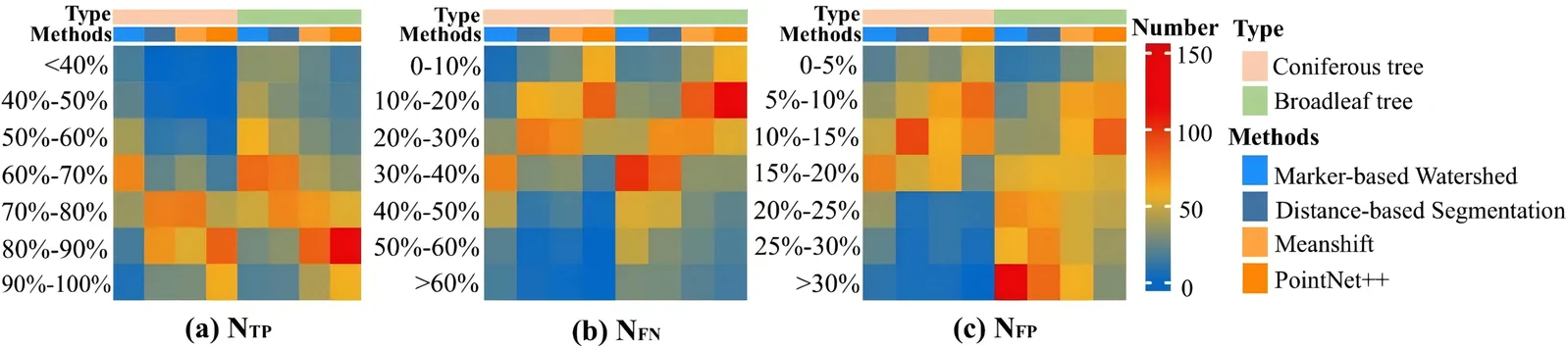
Evaluation of tree segmentation quality for different methods on the test plots. **(a)** True positives (NTP), **(b)** false negatives (NFN), and **(c)** false positives (NFP). The heatmaps focus on the three traditional methods and the proposed CA-IRM-PointNet++.

## 5 Discussion

### 5.1 Analysis of semantic segmentation in PointNet++

Due to the dominance of in mixed coniferous and broadleaf forest in the study area, tree species exhibited diverse morphologies. As a result, identifying tree points from different forest types using only coordinate information was insufficient. To improve classification accuracy, this study utilized true-color laser point clouds. The information of RGB, intensity, and echo counts was added to the standard (X, Y, Z) data to construct a semantic segmentation dataset with multi-channel feature information. The results showed that the network had stronger segmentation capabilities for ground points when using only coordinate information, achieving an mIoU of 88.81%. After incorporating additional features, the identification capability of tree point clouds improved significantly. Compared to the algorithm that used only coordinate data, the segmentation accuracy of PointNet++ improved by approximately 7.98% for coniferous trees and 6.27% for broadleaf trees. These results demonstrated that the inclusion of additional feature information enhanced the semantic segmentation capabilities of deep learning networks. Previous studies also supported this finding. For example, Ning et al. (2022) constructed a point cloud dataset that included XYZ information and six local features. Then, the PointNet network was used to perform semantic segmentation for forest point cloud. Their results showed that adding local features improved the overall segmentation accuracy by approximately 5%, while the IoU of tree and non-tree point clouds increased by approximately 13.5% and 5.5%, respectively. It was shown that local point cloud information can effectively enhance the semantic segmentation capabilities of networks. Similarly, Shin et al. (2022) demonstrated that multiple echo information from LiDAR can be applied to extract specific features. The echo signals generated by scanning trees were more abundant compared to other objects. Therefore, efficient differentiation of tree and ground points can be achieved by using multiple echo features. Lv et al. (2021) evaluated various feature descriptors for the classification performance of tree point clouds using the PointNet++ network. When using only point coordinates as input, the classification accuracy was 72.7%, significantly lower than the accuracy achieved when using additional feature descriptors. The convex hull-based descriptor achieved the highest accuracy (86.6%) by effectively capturing tree canopy shapes while ignoring less relevant details such as branches and leaves. Thus, it serves as an effective feature for tree point clouds classification.

The original PointNet++ network did not adequately consider the importance and correlation of different channels, limiting its semantic segmentation performance on multi-channel datasets. To address this issue, the channel attention mechanism was introduced in this study. By adaptively learning the weights of different channels, the network focused on the most representative channels, enhancing its discriminative power. The experimental results showed that the introduction of the channel attention mechanism can more effectively capture key information in the channels. Compared to the original PointNet++ network, the improved network significantly improved the accuracy of point cloud feature extraction and semantic segmentation (OA = 93.91%, mIoU =90.49%). Moreover, the convergence speed of the model is also a critical concern. The addition of multi-feature significantly increased computational complexity, which may negatively impact overall performance. Therefore, an IRM module combining inverted residual design and MLP structure was introduced in this study. The IRM module was intended to maintain feature extraction capability while keeping the computational cost manageable. Jing et al. (2021) proposed SE-PointNet++, a network architecture based on multispectral LiDAR data. This model incorporated the Squeeze-and-Excitation (SE) module which mainly included the channel attention mechanism and the adaptive recalibration. The results showed that embedding the SE module into PointNet++ could enhance important channels, improve feature saliency, and achieve better semantic classification. Through testing on the Titan multispectral LiDAR test dataset, the SE-PointNet++ architecture achieved an overall accuracy of 91.16% and an average IoU of 60.15%. Wang et al. (2023) introduced key points and a channel attention mechanism into the PointNet model to construct the KCA-PointNet deep learning improved network. By performing semantic segmentation on point cloud data generated from oblique photography, it was demonstrated that the KCA module improved segmentation accuracy for buildings, vegetation, and cars by approximately 3%. This enhancement not only eliminated redundant channel information but also reduced the possibility of losing features during point cloud data sampling, effectively improving the accuracy of point cloud data extraction and segmentation efficiency.

### 5.2 Analysis of individual tree segmentation accuracy obtained by different algorithms

In mixed coniferous and broadleaf forest, the diversity of tree species and the complexity of the environment make effective tree segmentation challenging. The PointNet++ deep learning algorithm can extract feature information from large volumes of raw point cloud data, and simplify the mixed forest plots into broadleaf and coniferous tree point clouds sets through semantic segmentation. This greatly reduced the complexity of subsequent individual tree instance segmentation. However, the high canopy closure in mixed forest often resulted in occlusion and overlap between trees, making it difficult to accurately determine tree locations. To address this issue, this study used the combination of local highest points and density maximum points to achieve precise tree localization, thereby mitigating the negative impact of tree overlap on segmentation results. Considering the morphological differences between coniferous and broadleaf trees, the Hough transform was used to automatically detect tree crown sizes and subsequently performed grid partitioning based on the detection results. This approach significantly improved segmentation accuracy by overcoming the challenge in traditional algorithms, which struggled to balance global and local optimal parameters in individual tree segmentation of mixed forest. To verify the feasibility of the proposed method in this study, three commonly used segmentation algorithms (Marker-based watershed, Distance-based segmentation, and Mean shift) were compared and analyzed. The results showed that the individual tree segmentation algorithm based on PointNet++ achieved the highest accuracy (r=0.836, p=0.849, F1 = 0.843). It also demonstrated strong performance in mixed coniferous and broadleaf forest plots, which was consistent with the results of other researchers. For example, Liu et al. (2023) investigated the influence of point cloud data source, tree species, and segmentation algorithms on the accuracy of individual tree crown segmentation. Their comparative experiments showed that the deep learning algorithm based on PointNet++ achieved better accuracy (F1 = 0.91). Similarly, Chen et al. (2021) compared the individual tree segmentation performance of the PointNet++ deep learning algorithm with two other traditional algorithms (watershed and region growing method). The results showed that the deep learning algorithm achieved 1% to 6% higher segmentation efficiency compared to the traditional algorithms.

In addition, the integrity of individual tree point clouds was introduced to evaluate the quality of tree segmentation. Traditional segmentation algorithms are often more susceptible to the effects of tree overlap and occlusion, leading to the wrong identification of tree locations and further exacerbating the misclassification of canopy point cloud. Among the four segmentation methods adopted in this study, the marker-based watershed algorithm had the lowest average integrity of tree point clouds, with only about 71%. This indicated that there were still a large number of individual trees with poor segmentation quality, which failed to meet the requirements of high-precision segmentation. To address the above issue, this study searched for potential tree location feature points by extracting local highest from the canopy point cloud. Subsequently, the method of density maxima method was used to accurately locate the individual trees, which significantly reduced point cloud misclassification caused by incorrect tree location estimation. The results indicated that this method achieved an optimal segmentation quality of 87.9%, representing an approximate 25% improvement in segmentation performance compared to traditional algorithms. The results further demonstrated the importance of accurate tree locationing in individual tree segmentation. Notably, the segmentation performance of coniferous and broadleaf trees exhibited significant differences. Coniferous tree crowns were typically compact and exhibited more regular shapes, reducing the possibility of large-scale overlap. In contrast, broadleaf tree crowns were larger and more irregular in shape, making them more prone to internal and inter-crown overlap, which complicated the segmentation of trees. Additionally, the morphological diversity of broadleaf tree crowns introduced greater randomness and variability during the feature extraction stage, which can further impact segmentation results.

### 5.3 Limitations and future research

The segmentation algorithm based on PointNet++ proposed in this study achieved promising results and alleviated point cloud misclassification caused by crown overlap and occlusion. However, the results showed that this issue was not fully resolved, particularly in the segmentation of broadleaf trees. This was primarily due to the high canopy density in mixed forest, which led to the larger crowns being more easily merged with point clouds from adjacent tree crowns. Even if the tree was correctly segmented, the missing crown point clouds can still affect the quality of individual tree point clouds. Therefore, we will develop a layered boundary extraction method for broadleaf tree crowns and focus on completing missing crown point clouds to enhance the usability of individual tree point clouds. Moreover, although each main model configuration was trained multiple times, the per-run metrics were not separately recorded. In future work, we will record per-run metrics and report mean ± standard deviation.In terms of the study area setting, the tree species in the mixed forest plots mainly include Larix gmelinii, Pinus koraiensis, Ulmus pumila, Betula platyphylla, Fraxinus mandshurica, Juglans mandshurica, and the uneven distribution of tree species may introduce noise into the segmentation results. Moreover, external validation on independent datasets from other regions or forest types was not feasible in this study. Future studies should conduct individual tree segmentation experiments with larger sample sizes to further validate the proposed method on more diverse datasets. To facilitate data collection and regular monitoring, most of the study plots were selected in flat areas with minimal topographic variation. However, topographic variations are associated with tree growth conditions, which may result in the same tree species exhibiting different characteristics. Therefore, it is essential to investigate the influence of topographic factors on individual tree segmentation performance.

In terms of deep learning, an IRM-only variant with multi-feature input was not trained in this study. Therefore, the individual contributions of the IRM module and the channel attention mechanism could not be fully separated, and the efficiency benefit of IRM was not quantified under identical conditions. In future work, we will train an IRM-only variant, report parameter counts and training/inference times under identical conditions to further evaluate the stability and efficiency of the proposed network. In addition, PointNet was added as a representative point-cloud deep learning baseline in this study. It is a seminal and widely used network for point cloud processing and is computationally feasible for comparison under identical experimental conditions. However, additional deep learning baselines such as DGCNN, KPConv, and RandLA-Net were not included due to computational and time constraints. Future work will incorporate more representative point-cloud deep learning architectures under the same data split and evaluation pipeline.

## 6 Conclusions

In this study, three-dimensional point cloud data with RGB information was obtained from the mixed coniferous and broadleaf forest plots using UAV LiDAR. To address the segmentation challenges in mixed forest, the PointNet++ deep learning algorithm was proposed to segment individual tree, and the accuracy and segmentation results were compared to traditional algorithms. The main conclusions were as follows:

1. Compared to the PointNet++ network that only included coordinate information, the OA of PointNet++ with multi-feature improved by 3.06% and mIoU improved by 5.64%. Furthermore, with the addition of the channel attention mechanism and IRM model, the convergence speed and training accuracy of the network were improved. The OA and mIoU values reached the optimal 93.91% and 90.49%, respectively.
2. The individual tree segmentation capabilities of four algorithms were tested, and the PointNet++ deep learning algorithm achieved the highest accuracy among the compared methods in this study (r=0.836, p=0.849, F1 = 0.843). From the segmentation accuracy of different tree types, coniferous trees generally outperformed broadleaf trees. Traditional algorithms exhibited a large number of over-segmentation occurrences, particularly in the segmentation of broadleaf trees, which was most pronounced in the marker-based watershed algorithm (p = 0.716). The segmentation method based on PointNet++ reduced the risk of wrong segmentation in mixed forest. The correct segmentation rate for coniferous trees reached 0.859, while for broadleaf trees, it was 0.821.
3. After evaluating the results of individual tree segmentation, the degree of integrity was further used to evaluate the quality of individual tree point clouds in this study. The proposed method based on PointNet++ achieved the highest segmentation quality of individual tree point clouds. Moreover, the average integrity of individual trees was over 85%, indicating that the algorithm has good stability.

Under the experimental conditions of this study, the proposed method based on the PointNet++ deep learning segmentation algorithm achieved good segmentation results in the studied mixed coniferous and broadleaf forest. The study results not only provided methodological support for improving the accuracy of individual tree segmentation in mixed forest but also represented basic data that can be used for extracting structural parameters of individual trees in forest.

## Data Availability

The raw data supporting the conclusions of this article will be made available by the authors, without undue reservation.
